# CD4^+^ T Cells Have a Permissive Effect on Enriched Environment-Induced Hippocampus Synaptic Plasticity

**DOI:** 10.3389/fnsyn.2018.00014

**Published:** 2018-06-13

**Authors:** Hadi Zarif, Salma Hosseiny, Agnès Paquet, Kevin Lebrigand, Marie-Jeanne Arguel, Julie Cazareth, Anne Lazzari, Catherine Heurteaux, Nicolas Glaichenhaus, Joëlle Chabry, Alice Guyon, Agnès Petit-Paitel

**Affiliations:** ^1^Université Côte d’Azur, CNRS, IPMC, Nice, France; ^2^Université Côte d’Azur, INSERM, IPMC, Nice, France; ^3^Université Côte d’Azur, INSERM, C3M, IPMC, Nice, France

**Keywords:** enriched environment, CD4^+^ T cells, hippocampus, brain plasticity, mice

## Abstract

Living in an enriched environment (EE) benefits health by acting synergistically on various biological systems including the immune and the central nervous systems. The dialog between the brain and the immune cells has recently gained interest and is thought to play a pivotal role in beneficial effects of EE. Recent studies show that T lymphocytes have an important role in hippocampal plasticity, learning, and memory, although the precise mechanisms by which they act on the brain remain elusive. Using a mouse model of EE, we show here that CD4^+^ T cells are essential for spinogenesis and glutamatergic synaptic function in the CA of the hippocampus. However, CD4^+^ lymphocytes do not influence EE-induced neurogenesis in the DG of the hippocampus, by contrast to what we previously demonstrated for CD8^+^ T cells. Importantly, CD4^+^ T cells located in the choroid plexus have a specific transcriptomic signature as a function of the living environment. Our study highlights the contribution of CD4^+^ T cells in the brain plasticity and function.

## Highlights

- CD4^+^ T cells play a major role in brain synaptic plasticity changes induced by an enriched environment in mice, as revealed by measurements of the CA hippocampus spinogenesis and glutamatergic synaptic function.

- CD4^+^ T cells do not affect EE-induced neurogenesis in the dentate gyrus (DG) of the hippocampus, by contrast to CD8^+^ T cells as previously showed.

- Brain CD4^+^ T cells sorted from dissected choroid plexus display a transcriptomic signature specific of the enriched environment as compared to standard environment as revealed by RNAseq.

## Introduction

Exposure to an enriched environment (including voluntary exercise, sensory stimulation, and social interactions) synergistically act on the whole body for instance by decreasing adiposity, increasing energy expenditure, favoring the immune responsiveness and increasing brain plasticity (van Praag et al., 2000; Sale et al., 2009; Cao et al., 2010). The hippocampus is a brain structure that is particularly susceptible to plasticity and involved in learning and memory regulation (Burgess et al., 2002; Opitz, 2014; Lazarov and Hollands, 2016; Zarif et al., in press). In a previous study, we have shown that raising mice for 4 weeks in an enriched environment (EE, consisting of a combination of complex inanimate and social stimulations) increases neurogenesis in the dentate gyrus (DG), induces changes in synapse morphology, and modifies synaptic plasticity in the hippocampus (Hosseiny et al., 2015). We found that more synapses were functional as the frequency of spontaneous and miniature post-synaptic currents (PPSCs) recorded on CA1 neurons was increased and that the field excitatory post synaptic potentials (fEPSPs) were larger, which was correlated to changes in LTP process.

One of the intermediaries through which EE can influence the brain is the immune system. In physiological conditions, stimulating environment of life concomitantly shapes the immune system and promotes the remodeling of neural circuits, the memory consolidation, the hippocampal neurogenesis and synaptogenesis. Whether these EE effects on the immune and the central nervous system are interdependent or unrelated remains to be clarified.

Kipnis et al. (2008) and Schwartz and Shechter (2011) recently suggested that circulating T cells play a crucial role in brain functioning, including brain plasticity and cognitive aptitudes. First experimental evidence for this notion was provided by demonstrating that mice devoid of T cells (such as severe combined immune deficiency, SCID mice, deficient in both T and B cells, and nude mice, deficient only in mature T cells), presented learning deficits compared to wild type mice, in the Morris water maze, a hippocampal-dependent spatial learning and memory task (Kipnis et al., 2004; Ron-Harel et al., 2008). SCID mice also exhibited impaired learning and memory in three other hippocampal-dependent tasks – the radial arm water maze, the water-free Barnes maze (Brynskikh et al., 2008) and the novel spatial arrangement recognition of familiar objects (Ron-Harel et al., 2008). We recently showed that CD8^+^ T cells phenotype is modified in EE relative to a standard environment (SE) leading to enhanced brain plasticity (Zarif et al., 2018). In the present study, we focused our investigations on the role played by CD4^+^ T cells in EE-related shaping of the hippocampus. We confirm here that CD4^+^ T cell populations affect EE-induced hippocampus synaptic plasticity and we further show that CD4^+^ T cell transcriptomic profile is specific of the EE housing as compared to SE. In addition, we demonstrate that CD4^+^ T cells act differently on EE-induced hippocampal plasticity, compared to CD8^+^ T cells, as they do not affect EE-induced hippocampal neurogenesis.

## Materials and Methods

### EE Breeding Conditions

C57BL6/J female mice (Janvier Labs, France) were exposed to EE from 4 weeks after birth (i.e. at weaning) for a 1, 2, 3 or 4-week period as described in (Zarif et al., 2018). A total of 164 C57BL6/J mice were used for this study. Age-matched mice (12–15) were housed in large-sized cages (9120 cm^2^; L × l × h: 120 cm × 76 cm × 21 cm) with nesting material, houses, running wheels, hammocks, scales, plastic toys and tunnels. Objects were changed twice a week. Mice in standard conditions (SE) were housed in medium-sized cages (666 cm^2^; L × l × h: 36 cm × 18.5 cm × 14 cm) with 5–6 mice/cage and without objects. All mice had access to tap water and standard lab chow (diet SAFE A04, 2900 kcal/kg) *ad libitum* and were housed in a 12-h light/12-h dark cycle at 22–23°C with 40–60% humidity. The animals analyzed in each experiment were randomized either in SE or EE.

All animal studies were carried out in accordance with French standard ethical guidelines for laboratory animals (Agreement No. 75-178, 05/16/2000) and the European Communities Council Directive of 24 November, 1986 (86/609/EEC), in compliance with the Institutional Animal Care and Use Committee of the University of Nice-Sophia Antipolis (permission number 010344.01) from the French “Ministère de l’Enseignement Supérieur et de la Recherche.” Formal approval to conduct the experiments described was obtained from the animal subjects review board of this institution and can be provided upon request. All efforts were made to minimize the number of animals used and their suffering.

### T Cell Depletion

To achieve selective T cell sub-population depletion, each 3-week-old female mouse (C57BL6/J) was injected ip with 0.5 mg depleting antibody: either anti-CD4 Ab Ig (rat IgG2b, clone GK1.5, Ref BE0003-1 from BioXCell or from hybridomas culture supernatant ATCC^®^ No. TIB-207^TM^), or with control isotype Ab (control IgG; clone LTF-2, Ref BE0090 from BioXCell).

Three days after the first injection, mice were placed in different housing conditions, SE or EE, where they received the second and third injections (0.3 mg), 10 days apart each. The control groups received control antibody at the same time. Eight days after the last antibody injection, the mice were sacrificed, spleens were harvested and immune cells prepared to control for the absence of CD4^+^ T cells by using flow cytometry with the anti-CD3 and anti-CD4 antibodies. We observed no depletion of CD4^+^ T cell in control Ab-injected mouse spleen, while around 98% depletion was observed with the anti-CD4 antibody (**Supplementary Figure S1**).

### Hippocampal Neurogenesis

We measured hippocampal neurogenesis in EE and control SE mice using intra-peritoneal injections of Bromodeoxyuridine (BrdU) (50 mg/kg, once a day for 5 consecutive days) followed by immunohistochemistry quantification of BrdU-stained cells in the hippocampus, according to (Heurteaux et al., 2006). Briefly, mice were euthanized with pentobarbital 24 h or 21 days after the last injection, perfused with ice-cold HBSS (pH7.4, 1 mg/mL EDTA) and fixed by 3.2% PFA through intra-cardiac perfusion. Brain tissue was rapidly removed and fixed in 3.2% paraformaldehyde (PFA) for 48 h. 40-μm thick serial sections of PFA-fixed brains were cut throughout the hippocampus on a vibratome (Microm). One of every six slices were collected for a total of eight to twelve, which were analyzed by immunohistochemistry staining using a monoclonal mouse anti-BrdU (1:7000; BD Biosciences). For BrdU chromogenic immunodetection, sections were incubated for 1 h in biotin-conjugated species-specific secondary antibodies (1:400, Vector Laboratories), followed by a peroxidase-avidin complex solution according to the manufacturer’s protocol. The peroxidase activity of immune complexes was visualized with 3,3′-Diaminobenzidine (DAB) staining using VectaStain ABC kit (Vector Laboratories). BrdU-labeled cells of granular and subgranular layers were counted in each section under a light microscope. The total number of BrdU^+^ cells counted per eight slices was multiplied by six to obtain the total number of BrdU^+^ cells per DG.

We also performed a double labeling with both the monoclonal mouse anti-BrdU (1:500; BD Biosciences) and a secondary donkey anti-mouse Alexa fluor 488 (Invitrogen) and the polyclonal rabbit anti-NeuN (1:1000, Millipore) coupled to a secondary donkey anti-rabbit Alexa fluor 594 (Invitrogen) antibodies to investigate newborn cells and identify those that were differentiated into neurons, following the protocol of Wojtowicz, Nature Protocols, 2006.

### Hippocampal Spinogenesis

Mice were deeply anesthetized with pentobarbital and perfused with 3.2 % paraformaldehyde (PFA). A single brain serial section (200 μm) was cut at the level of the hippocampus on a vibratome (Microm) approximately between bregma 2.30 and 2.50 mm. from the same mice as those used for the neurogenesis experiments. The slices were then stored in 0.1 % (wt/vol) NaN_3_ in PBS at 4°C until microinjected with the fluorescent dye Alexa Fluor 568 (Thermo Fisher Scientific) by iontophoresis coupled with pressure ejection using micropipettes with high tip resistance (15-20 MΩ). The slices were then mounted using a Vecta-Shield mounting medium and imaged within a few days.

Stacks of images from segments of 45-μm long dendrites were obtained through a 63X/1.4 NA objective on an LSM780 laser-scanning confocal microscope (Carl Zeiss, Le Pecq, France). A detailed morphometric analysis of the spines was first performed using an in-house macro-program from ImageJ software (Rasband, W.S., ImageJ, US National Institutes of Health, Bethesda, Maryland, USA, http://imagej.nih.gov/ij/, 1997-2012). Briefly, after maximal projection of the images of each stack and segmentation to produce a binary image, the skeleton of the dendritic tree was analyzed and its length measured. The number of intersections and spines were determined and after binary thinning, the length of the principal axis was measured. These images were subsequently analyzed with NeuronStudio software and spines were classified into “thin,” “mushroom,” and “stubby” according to Peters and Kaiserman-Abramof (1970) using the following parameters: the maximum and minimum spine heights were set at 3.5 and 0.5 μm, respectively. Minimum stubby spine was set at 22 voxels. In each group, we used 66–89 segments of dendrites from 23–30 neurons from 5–6 mice (**Supplementary Figure S2**).

### Hippocampal Electrophysiology

#### Acute Brain Slices

Mice were deeply anesthetized with halothane then decapitated and brains were immediately placed into ice-cold gassed medium (95% O_2_/5% CO_2_) containing (in mM): 125 NaCl, 2.5 KCl, 1 MgCl_2_, 0.4 CaCl_2_, 1.25 NaH_2_PO_4_, 26 NaHCO_3_, and 25 glucose. Coronal slices of hippocampus (350 μM thick) were cut with an HM650V vibratome (Microm, Walldorf, Germany) and placed in a holding chamber at 34°C for 1 h. Slices were then transferred into a Phosphate Bicarbonate Buffer Saline (PBBS) composed of (in mM): 125 NaCl, 2.5 KCl, 1 MgCl_2_, 2 CaCl_2_, 1.25 NaH_2_P0_4_, 26 NaHCO_3_ and 25 glucose, pH 7.4 when bubbled with 95% O_2_ and 5% CO_2_.

#### Patch Clamp Technique

CA1 pyramidal neurons were patch-clamped in the whole-cell configuration. This technique allowed for recording of currents from the whole surface of a single neuron in the living slice while still connected with the rest of the neuronal network. Using pipettes (2–8 MΩ) filled with a cesium chloride (CsCl) solution supplemented with *N*-(2,6-Dimethylphenylcarbamoylmethyl)triethylammonium bromide (QX314, a sodium channel blocker to block action potentials) we recorded the glutamatergic excitatory post-synaptic currents (EPSCs) which were pharmacologically isolated using the GABA_A_ receptor antagonist bicuculline (10 μM) in the bath solution. We recorded both spontaneous (without the sodium channel inhibitor tetrodotoxin TTX) and miniature (in the presence of 2 μM TTX) EPSCs. Three-minute-long recordings were used to determine the properties of the spontaneous events. 3–16 neurons were recorded in 2–5 mice in each group. The same mice were used both for patch-clamp and LTP experiments using two different electrophysiology setups simultaneously.

#### Long-Term Potentiation (LTP)

Hippocampal slices were placed under a Nomarski microscope (Zeiss, Germany) equipped with an infrared video camera (Axiocam, Zeiss) in a recording chamber superfused at a flow rate of 1 mL.min^-1^ with oxygenated PBBS. Pictures were taken using a digital camera (Axiocam, Zeiss) connected to image-acquisition software (Axiovision, Zeiss). Recordings were made at room temperature (20–25°C) using an Axopatch 200B (Axon Instruments, Foster City, CA, United States) connected via an interface (Digidata 3200) to a computer running pClamp (Axon Instruments). At the beginning of each recording, a tungsten bipolar stimulating electrode was positioned at the stratum radiatum for stimulation of the Schaffer collateral projections to CA1, using a stimulator (STG4002 Multichannel systems) connected to the computer. Field potentials in the dendritic tree of CA1 neurons were recorded with pipettes (made from borosilicate glass capillary (Hilgenberg, Masfeld, Germany) with resistance of 3–6 MΩ when filled with extracellular solution). The intensity of stimulation was adjusted in each experiment to evoke about 50% of the maximal field potential amplitude without appreciable population spike contamination. Low-frequency stimulation (0.1 Hz) was applied to the Schaffer collaterals to establish a stable baseline (for 20-30 min) of the excitatory post synaptic potential (EPSP) slope, after which LTP was induced by high-frequency stimulation (HFS; 100 Hz/1 sec), followed by the initial low frequency stimulation. To analyze the time course of the EPSP slope, the recorded fEPSP data were routinely averaged over 1 min (*n* = 6). Successful induction of LTP was obtained when the post-HFS EPSP exceeded that seen before HFS and was maintained for at least 40–60 min. 3–5 mice were used per group.

#### Data Analysis

Voltage clamp data were digitized at 0.5 kHz using a Digidata interface coupled to a microcomputer running p-Clamp 9 (Axon Instruments). Currents were digitally filtered at 1–3 kHz. Average data were expressed as mean ± SEM, *n* = number of neurons that were recorded. Statistical significance between groups was calculated using the Student *t*-test, the ANOVA followed by the Fisher test, or the Kruskal–Wallis followed by the Dunn’s test and were considered significant at ^∗^*p <* 0.05, ^∗∗^*p <* 0.01 and ^∗∗∗^*p <* 0.001 using a statistical software package (SigmaStat 2.03, Jandel Sci and Graph Prism software). Cumulative histograms were compared by Kolmogorov-Smirnov analysis using Clampfit (Axon Instruments), with an equal number of events for each group. Significant differences between two groups of data were determined using a Mann–Whitney test for non-parametric data.

### RNA Isolation and Quantitative Polymerase Chain Reaction (qPCR)

#### RNA Isolation

Total RNA from whole hippocampus, DG and CA, dissected as described in (Hagihara et al., 2009) was isolated using the Trizol^®^ RNA extraction kit (Invitrogen) according to the manufacturer’s recommendations followed by a RQ1 DNAse (Promega) treatment. First-strand cDNAs were synthesized from 2 μg of total RNA with 200 U of SuperScript III reverse transcriptase (SuperScriptIII, Invitrogen) in the appropriate buffer in the presence of 25 μg/mL random primers, 0.5 mM desoxyribonucleotide triphosphate mix, 5 mM dithiothreitol, 40 U RNAsin (Promega). The reaction was incubated 5 min at 25°C, 50 min at 50°C then inactivated 15 min at 70°C.

#### qPCR

Quantitative PCR was performed using the SYBRgreen method (Roche) with a LightCycler 480 sequence detector system (Roche Diagnostics). β-actin and GAPDH were used as housekeeping genes for normalization. Primers were purchased from QIAGEN (QuantiTect primer assay, QIAGEN).

The following primers were used in **Table 1**:

**Table 1:** List of primers used for qPCR.

Gene	Cat. no.	Gene	Cat. no.
		
		DG and CA genes	
β-actin	QT01136772		
Bdnf	QT00097118	Dsp	QT00321496
Dlg4	QT00121695	Tdo2	QT00150409
Homer1	QT00129983	Tyro3	QT00197659
Slc17a7	QT00148841	Meis1	QT00172557
Syn1	QT00171206		
Syn2	QT00152698		
Syp	QT01042314		
Syt1	QT00167300		


**Table 2:** Number of mice, dendrites and spines used in **Figure 2** for the different groups.

	SE control	EE control	SE CD4^+^	EE CD4^+^
Number of mice	6	5	5	6
Number of dendrites	69	66	82	89
Number of spines	999	1374	1319	1505


**Table 3:** 12 genes exhibiting the most significant differences between Enriched and Standard Environments in CD4^+^ T cells from choroid plexus tissue.

Name	baseMean	log2FoldChange	pvalue
Rev1	2.820	2.220	0.001
Pole3	2.615	2.132	0.003
Hist1h2ae	3.832	2.062	0.007
Hist1h4m	2.170	2.052	0.004
Snx18	2.286	2.006	0.002
Wls	3.939	1.890	0.001
Plekha1	3.743	-1.740	0.003
Tmed8	2.174	-1.775	0.004
Atp7a	2.297	-1.938	0.002
Wdr13	2.516	-2.046	0.005
Diap2	2.747	-2.459	0.000
Prdm4	2.131	-2.642	0.000


*Candidate Database of Essential Genes (DEG) selected using the following criteria: *p* value < 0.01, abs(log2FoldChange) > 1, baseMean > 2.*

To confirm that the dissected tissue was DG, we measured specific gene expression (Dsp, Tdo2 and Ammon’s horn enriched genes, Tyro3, Meis1) using qPCR according to (Hagihara et al., 2009). Gene expression was analyzed following the delta delta Ct method.

#### Statistical Analysis

For each gene, we first performed a non-parametric Kruskal–Wallis test on ΔCt values followed by exact two-sample Fisher–Pitman permutation tests comparing SE to EE at 3W and 4W, and in each group (SE or EE) comparing 3W to 2W and 4W to 2W. The *p* values were corrected using the FDR method of Benjamini–Hochberg for a total of eight comparisons per gene.

#### Immune Cells Staining, Flow Cytometry, and Cell Sorting

Staining of brain immune cell surface antigens was performed as previously described (Cazareth et al., 2014). Briefly, Fc receptors were blocked with a purified rat anti-mouse CD16/CD32 (2.4G2, Fc block) antibody (BD Biosciences). Cells were incubated with the appropriate combination of conjugated antibodies: anti-CD11b-PerCP-Cy5.5, anti-CD45-APC-Cy7, anti-CD3-FITC, anti-CD8-PE-Cy7 or anti-CD8-PE, anti-CD4-BV510 or anti-CD4-PB (BD Biosciences) antibody for 30 min. Phenotype analyses of cells were performed in a flow cytometer (LSR II Fortessa, BD). Immune cells were sorted on a Becton-Dickinson FACS Aria III.

### RNA Sequencing

#### Cell Sorting

Mice were deeply anesthetized with a lethal injection of pentobarbital and transcardially perfused with ice-cold HBSS containing 1 mg/ml EDTA. Choroid plexus were collected on two independent series of experiments. Splenocytes were suspended by mechanic dissociation and immune cells prepared after ACK treatment to remove red blood cells. Choroid plexus samples were dissociated in PBS containing 3 mg/ml collagenase D (Roche Diagnostics), incubated 20 min at 37°C, filtrated through a cell strainer size 70 μm then centrifuged for 10 min at 1000 rpm. Cell pellets containing choroid plexus immune cells were collected, washed with PBS containing 0.5% BSA and 2.5 mM EDTA and labeled. Staining of immune cells from choroid plexus was performed as previously described (Cazareth et al., 2014). Immune cells were identified according to the surface antigen labeling of anti-CD45, anti-CD11b, anti-CD3, anti-CD4 and anti-CD8 conjugated antibodies (BD Biosciences). CD45^+^ CD11b^-^ CD3^+^ CD4^+^ and CD45^+^ CD11b^-^ CD3^+^ CD8^+^ cells were sorted by FACSAria cell sorter (BD Biosciences)

#### cDNA Preparation

Cells were directly collected into 0.2 mL tube stripes in a lysis reaction with a final volume of 13.5 μL according to Arguel et al. (2017), all incubations steps occurred in a Veriti thermal cycler (Applied Biosystem). Reverse transcription (RT) mix was added during incubation at 10°C immediately following lysis, tubes were vortexed and centrifuged briefly and put back in thermocycler for RT incubations steps. Template switching oligonucleotide (TSO) was designed as per Picelli et al. (Picelli et al., 2014), with a LNA on the 3′ extremity 5′-GCA ATG AAG TCG CAG GGT TGN NNN HHH HrGrG lnG-3′.

For CD4^+^ T cells from the choroid plexus, the number of cells varied from 2 to 20 cells per sample, thus the entire RT volume was added to the PCR amplification reaction, leading to 31.5 μl of RT in a final volume of 83 μl, 60 μM barcode primer, 0.6 μM biotinylated PCR primer.

#### Library Preparation

All 48 barcoded cDNA samples were pooled and 20 ng used as a template for the Ion Proton sequencer tagmentation protocol as described (Arguel et al., 2017).

#### Sequencing

Libraries were sequenced on a Proton Ion PI^TM^ Chip V3 (Thermo) generating 38 M reads for the choroid plexus. Single-end reads were processed with a custom analysis pipeline. The first step was removing the 3p adaptor “CTGTCTCTTATACACATCT” and the front adaptor sequence “AAGTCGCAGGGTTG” using cutadapt (version 1.2.1). We required, immediately after the front adaptor sequence, an 8-base pattern in accordance with the UMI design N4H4 (N = ATCG, H = ATC) followed by a stretch of “GGG”. Reads without those specifications were discarded from further analyses to avoid artifactual new molecule production due to creation of pseudo UMI sequences related to sequencing errors, especially indels that are frequent in Ion Torrent reads. Reads with a template sequence length under 50 bases were also discarded. This filtration process removed 32% and 34% of the total amount of reads. Mapping of the cDNA sequences was then done with STAR_2.4.0a versus mm10 mouse genome build using RNA-seq Encode recommendations. For molecule counting based on UMI counts, we used the Dropseq Core Computational Protocol version 1.0.1 (dropseq.jar) from McCarrol (Macosko et al., 2015) using GTF gene model from Ensembl release GRCm38.83. The Digital Expression function of dropseq.jar was used with default parameters (edit distance = 1) to produce a matrix of molecule counts that were used in the subsequent statistical analysis.

#### Statistical Analysis and Biological Theme Analysis

Choroid plexus samples were analyzed separately. Quality control of RNAseq data was performed with in-house R scripts. Purity of biological samples was verified by inspection of CD4 expression levels. CD4 samples with non-zero expression levels for CD8a and CD8b were considered of poor quality and excluded. Low abundance genes were filtered out, then sequencing depth normalization and differential expression analysis where carried out using the Bioconductor package DESeq2 (Love et al., 2014). *P*-values were adjusted for multiple testing using the Benjamini–Hochberg procedure, which controls the false discovery rate. No gene reached statistical significance at the 0.05 level after adjustment. For choroid plexus samples, mild differential expression was observed between enriched and standard environments and candidate differentially expressed genes were selected based on a nominal *p*-value < 0.05. Heatmaps were generated using the R package pheatmap (Pretty Heatmaps V 1.0.8, https://CRAN.R-project.org/package=pheatmap).

Canonical pathways and molecular function analyses were carried out using Ingenuity Pathway Analysis (IPA, Qiagen Bioinformatics). Gene Set Enrichment Analysis was performed using GSEA, by comparing the modifications observed in EE vs. SE to modifications observed in the same subcellular population in the immune set of data (C7) or a larger set of data (C2). Pathways with a normalized *p* value < 0.05 were considered as enriched.

### Accessibility

The experimental data have been deposited in the NCBI Gene Expression Omnibus (GEO) under accession number GSE100584 for spleen serie and GSE100586 for plexus chorod serie.

### Drugs

APV [(2R)-amino-5-phosphonovaleric acid, an *N*-methyl-D-aspartate (NMDA) receptor antagonist] and 6-cyano-7-nitroquinoxaline-2,3-dione (CNQX), a α-amino-3-hydroxy-5-methyl-4-isoxazolepropionic acid receptor (AMPA)/Kainate receptor antagonist, QX314, TTX and bicuculline were from Sigma Aldrich, France.

### Data Analysis

Two-way ANOVA, followed by a post-hoc Tukey’s multiple comparison tests were performed when data followed the normality Shapiro–Wilk test. Otherwise, the non-parametric ANOVA Kruskal-Wallis test was used followed by a *post hoc* + Dunn’s test.

Voltage clamp data were digitized at 0.5 kHz using a Digidata interface coupled to a microcomputer running p-Clamp 9 (Axon Instruments). Currents were digitally filtered at 1–3 kHz. Average data are expressed as mean ± SEM, *n* = number of neurons that were recorded. Statistical significance between groups was calculated using the Student *t*-test, the ANOVA followed by the Fisher test, or the Kruskal–Wallis followed by the Mann–Whitney test and were considered significant at ^∗^*p* < 0.05, ^∗∗^*p* < 0.01 or *p* < 0.02 as mentioned, and ^∗∗∗^*p* < 0.001 using a statistical software package (SigmaStat 2.03, Jandel Sci). Cumulative histograms were compared by Kolmogorov-Smirnov analysis using Clampfit (Axon Instruments), with an equal number of events for each group. Significant differences between two groups of data were determined using a Mann–Whitney test for non-parametric data.

## Results

### CD4^+^ T Cells Are Required for the EE-Induced Hippocampal Synaptic Plasticity

#### CD4^+^ T Cell Depletion Does Not Affect EE-Induced Neurogenesis

**FIGURE 1:**
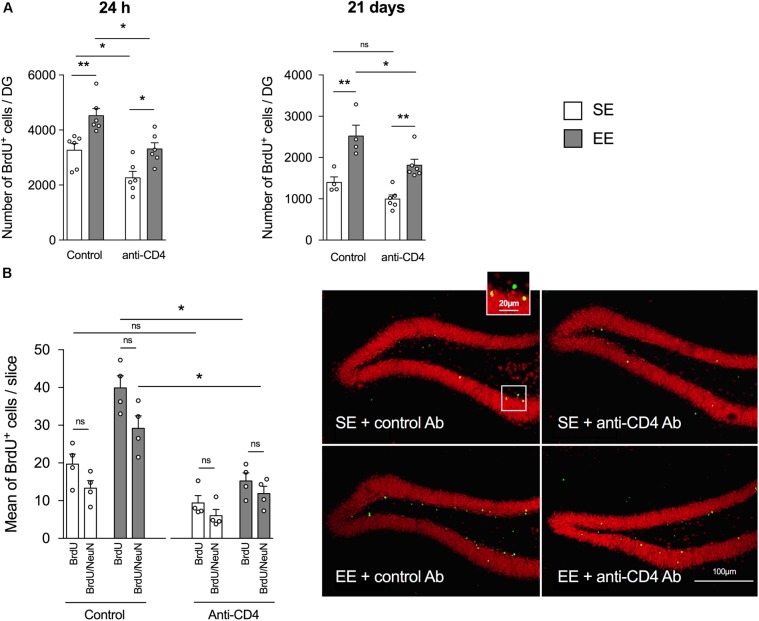
CD4^+^ T cell depletion affects EE-induced neurogenesis in dentate gyrus of the hippocampus from SE and EE mice. **(A)** Chromogenic immunodetection of newborn BrdU^+^ cells measured in the DG 24 h after BrdU injection (left) or 21 days after the first BrdU injection (right) in mice raised in SE (white) or EE (gray). Mice were treated either with the depleting anti-CD4 or the control isotype antibodies (*n* = 4–6 mice per group). **(B)** Right Panel: confocal micrographs of the DG from control (left) or CD4^+^ T cell-depleted (right) mice housed in SE (top) or EE (bottom) showing the labeling of BrdU^+^ (green) and NeuN^+^ (red) cells. Left Panel: Histogram showing the mean number per slice (two slices labeled per hippocampus, *n* = 4 mice per group) of BrdU^+^ cells and BrdU^+^ NeuN^+^ cells.

As previously shown in the enrichment model set up in the laboratory (Hosseiny et al., 2015; Zarif et al., 2018), 4 weeks of housing in EE significantly increased hippocampal neurogenesis relative to SE in control-antibody-injected mice (**Figures 1A,B**), both when measured 24 h after injection (proliferation, **Figure 1A** left panel) and 21 days post-BrdU injection (proliferation + survival, **Figure 1A**, right panel). Most BrdU^+^ labeled cells in the DG were neurons, as shown by the double labeling with NeuN (**Figure 1B**) and doublecortin (DCX, **Supplementary Figure S3A**). A double labeling with Iba1 showed that less than 5% of BrdU^+^ cells were microglia (**Supplementary Figure S3B**). It is noteworthy that the number of BrdU^+^/NeuN^+^ of cells (differentiated granular neurons) in the DG was significantly higher in the control EE than the control SE group (**Figure 1B**).

The depletion of CD4^+^ T cells decreased the global number of BrdU^+^ cells measured both 24 h and 21 days after injection of BrdU (**Figure 1A**, left and right panels, respectively). However, CD4^+^ T cell depletion failed to blunt the increase in BrdU^+^ labeled cells induced by EE, both at 24 h and 21 days post BrdU injection. Therefore, basal neurogenesis mechanisms but not EE-induced neurogenesis mechanisms appear to be CD4^+^ T cells-dependent.

#### CD4^+^ T Cell Depletion Affects EE-Induced Synaptic Plasticity in CA1

**FIGURE 2:**
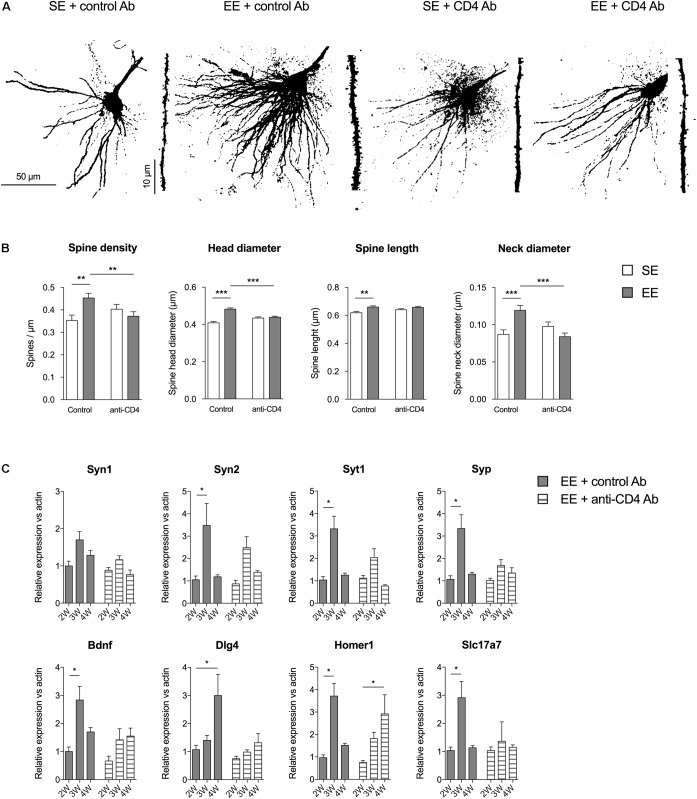
CD4^+^ T cell depletion affects spine density and morphology in pyramidal neurons of CA1. **(A)** Representative photomicrographs of CA1 pyramidal neurons of the hippocampus labeled with Alexa Fluor 568 from mice raised in SE or EE and treated with control or anti-CD4 Ab. **(B)** CD4^+^ T cell depletion modifies EE-induced spinogenesis and dendritic spine morphology in CA1 neurons. Spine characteristics (number of spines/μm, mean spine head diameter, mean spine neck diameter, mean spine length) were determined using NeuronStudio on segments of dendrites of 45–50μm at the second basilar dendrites of CA1 neurons labeled with Alexa Fluor 568. Bars are means ± SEM. SE is in white and EE is in gray. ^∗∗^*p* < 0.01 and ^∗∗∗^*p* < 0.001. Mann–Whitney test for comparison between groups was performed (**Table 2**). **(C)** CD4^+^ T cell depletion blocks EE-induced expression increases for synaptic factor genes in the CA of the hippocampus. CA were micro-dissected from hippocampus tissue and transcript abundance of neurotrophic genes was determined by RT-qPCR after different durations of housing (2W, 3W, and 4W) in SE or EE conditions. Bars represent the mean expression levels ± SEM of the 2^-ΔΔ*Ct*^. For each gene, we first performed a Kruskal–Wallis test on the ΔCt values followed by an exact two-sample Fisher-Pitman permutation test comparing SE to EE at 3W and 4W, and comparing in each group (SE or EE) 3W to 2W and 4W to 2W. The *p* values were corrected using the FDR method of Benjamini–Hochberg for a total of eight comparisons per gene. ^∗^*p* < 0.05 and ^∗∗^*p* < 0.01, *N* = 6 for each group.

We evaluated the effects of CD4^+^ T cell depletion on the number and types of spines of basilar dendrites from pyramidal CA1 neurons (**Figure 2**). We found that the enhanced spine density induced by EE in control conditions was abolished in mice depleted of CD4^+^ T cells. CD4^+^ T cell depletion also affected the variations in spine shape observed in mice raised in EE as compared to SE. For instance, the increases in spine length, head and neck diameter induced by EE in control Ab injected mice were absent in CD4^+^ T cell depleted mice. In conclusion, CD4^+^ T cell depletion affects the EE-induced spinogenesis modifications in pyramidal CA1 neurons of the hippocampus.

These effects in spine morphology were accompanied by variations in the expression of genes coding for synaptic proteins in CA. We micro-dissected the CA of the hippocampus of mice raised for 2, 3, and 4 weeks in SE or EE, and measured the expression of synaptogenesis-related genes by qPCR. We quantified the gene expression of several key presynaptic components (synapsin 1 and 2, synaptophysin and synaptogamin), the neurotrophic factor Bdnf, and synaptic proteins particularly expressed at the glutamatergic synapse (Dlg4, coding for the PSD95 scaffolding protein located in neural postsynaptic densities that associates with NMDA receptor NR2 subunits; homer1, encoding for Homer 1, a protein concentrated in postsynaptic structures; and Slc17a7 coding for the glutamate transporter Vglut1) (**Figure 2C**).

In EE housing conditions, the profile of kinetic expression of the mRNA of interest was quite different after depletion of CD4 (**Figure 2B**). Mice injected with control antibodies and raised in EE (EE + control Ab group) exhibited a significant increase in expression of genes coding for Syn2, Syt1, Syp, Bdnf, Homer1, and Slc17a7 at 3 weeks of EE housing, and in expression of Dlg4 gene at 4 weeks. CD4^+^ T cell depletion reduced or abolished these increases in expression of Syn2, Syt1, Syp, Bdnf, Dlg4, and Slc17a7 that had been observed in EE, and delayed the peak of expression of Homer1 to 4 weeks. Therefore, CD4^+^ T cells are required for the changes in gene expression induced by EE for those genes that encode proteins involved in the formation, stability and function of synapses.

#### CD4^+^ T Cell Depletion Affects EE-Induced Changes in Glutamatergic Transmission and LTP at the CA3-CA1 Synapses

**FIGURE 3:**
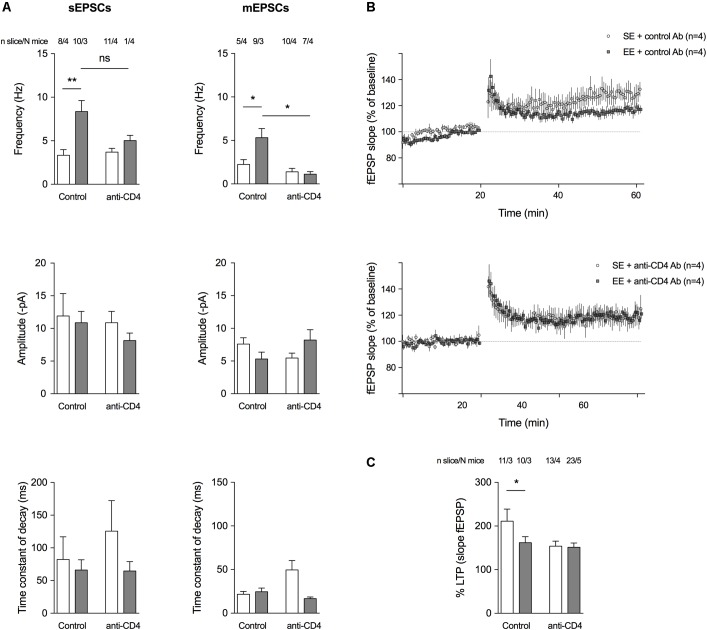
CD4^+^ T cell depletion affects spontaneous and miniature activities on pyramidal CA1 neurons and LTP at the CA3-CA1 synapse. **(A)** Histograms representing event frequency, event amplitude, and the time constant of decay of spontaneous (left) and miniature (right) EPSCs recorded in pyramidal CA1 neurons by patch clamp in a whole cell configuration. SE is in white, and EE is in gray. Number of neurons and mice used are indicated at the top. C ^∗^*p* < 0.05, ^∗∗^*p* < 0.01, Mann–Whitney test for comparison between groups was performed. **(B)** Field excitatory postsynaptic potentials (fEPSPs) recorded in the CA1 region of the hippocampus in response to a stimulation of Schaffer collaterals every 30 s. Stimulating intensity was chosen to trigger a fEPSP of 50 % of the maximum response. After a stable baseline of 20 min, the high-frequency stimulation (HFS) protocol was applied (100 Hz during 1 s). Then, the 30 s stimulation with the same intensity was restarted and a potentiation of the response to the stimulation was observed as expected, to 125–300 % of the initial response. Curves show the time course of the slope amplitude of the EPSPs for an average of representative slices of each group of mice, a group raised for 8 weeks in SE and a group raised for 4 weeks in EE from 4 weeks of age (*N* = 4 each). LTP was established in both groups after the HFS, but its magnitude was less pronounced in the group of mice raised for 4 weeks in EE. **(C)** Histograms showing the mean amplitude of LTP ± SEM measured 40 min after HFS according to the different conditions. SE is in white and EE is in gray. Number of slices and mice used are indicated at the top. ^∗^*p* < 0.05. Mann–Whitney test for comparison between groups was performed.

Since CD4^+^ T cell depletion affects the morphology of spines and the expression of genes encoding proteins involved in synaptic function, we investigated whether it also altered spontaneous glutamatergic activity (**Figure 3A**). We used a patch-clamp technique in the presence of 10μM bicuculline (in order to block GABA_A_ IPSCs) to record both spontaneous EPSCs (sEPSCs) and action-potential independent miniature EPSCs (mEPSCs, in the presence of 2 μM TTX) from SE or EE mice depleted of CD4^+^ T cells and compared to control-antibody-treated mice. CD4^+^ T cell depletion blocked the increase in sEPSCs and mEPSCs induced by EE (**Figure 3A**). As previously described (Hosseiny et al., 2015; Zarif et al., 2018), in EE conditions, groups of mice treated with control Ab had a lower magnitude LTP compared to those of same age under SE conditions (**Figures 3B,C**). When CD4^+^ T cells were depleted, there was no longer a difference in LTP levels between SE or EE groups of mice (**Figures 3B,C**) suggesting that CD4^+^ T cells might influence LTP.

Overall, CD4^+^ T cells are required for EE-induced hippocampal plasticity. Depletion of CD4^+^ T cells has differential effects on neurogenesis and synaptic activity in SE and EE conditions, suggesting that CD4^+^ T cells from SE- and EE-housed mice might be different.

### CD4^+^ T Cells Are Modified in EE as Revealed by RNAseq Experiments on CD4^+^ T Cells Sorted From Choroid Plexus Tissue

In a previous study, we found no difference in the characteristics of peripheral CD4^+^ T cells between SE and EE conditions (number, percentage among CD3^+^ cells, ratio of CD4^+^/CD8^+^ T cells, proliferation and cytokine production of peripheral CD4^+^ T cells sorted from spleen and *in vitro* stimulated for 72 h with CD3/CD28 or 4 h with PMA/Iono) (Zarif et al., 2018).

In order to investigate possible differences between the genes expressed in cerebral CD4^+^ T cells from mice raised in SE or EE, we conducted RNAseq experiments on CD4^+^ T cells sorted from choroid plexus tissue. We used a technique adapted from single-cell RNAseq that we previously developed, allowing the use of a very small number of cells (2 to 20 cells) sorted from the choroid plexus.

**FIGURE 4:**
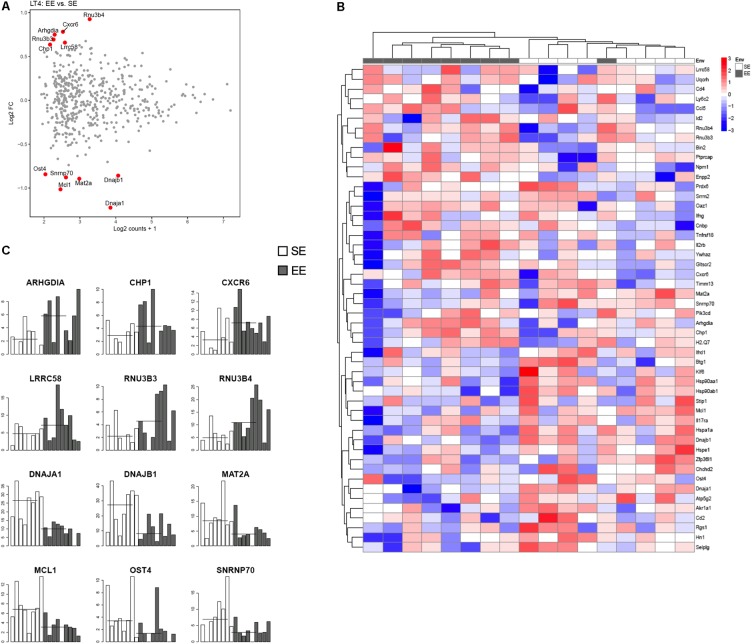
Transcriptomic analysis of CD4^+^ T cells from choroid plexus of mice raised in EE and SE. **(A)** MA-plot for the comparison between enriched and standard environments for CD4^+^ T cells from choroid plexus tissue. X-axis: average gene expression levels measured as log2 UMI counts + 1. Y-axis: log2 Fold-Change between EE and SE. Red dots: differentially expressed genes, selected based on a combination of *p* value < 0.01, abs(log2 Fold-Change) > 1 and average expression > 2. **(B)** Gene expression levels of the 12 genes exhibiting the most significant differences between Enriched and Standard Environments in CD4^+^ T cells from choroid plexus tissue. Data are expressed as UMI counts (**Table 3**). **(C)** Heatmap of the 50 most differentially expressed genes when comparing plexus choroid CD4^+^ T cells from enriched and standard environments. Expression levels are gene centered, so that all genes have an average expression level equal to zero. Hierarchical clustering of the samples and genes used Euclidean distance and complete linkage. Note: the differences in gene expression levels between EE and SE are small for this condition, and the hierarchical clustering is not able to separate EE and SE samples as clearly as in the other conditions.

**Figure 4** shows representative histogram of the expressed genes by the CD4+ cells from the choroid plexus. We found that the expression of a discrete set of genes differed significantly between CD4^+^ T cells from mice raised in EE vs. SE. Indeed, we found 67 genes (43 up, 34 down) with varying expression, *p* value < 0.01, abs(log2 Fold-Change) >1 and average expression >2. Among them, CXCR6, IFNγ, and CCL5 showed a greater expression in EE vs. SE (genes are listed in Zarif et al., 2018).

**FIGURE 5:**
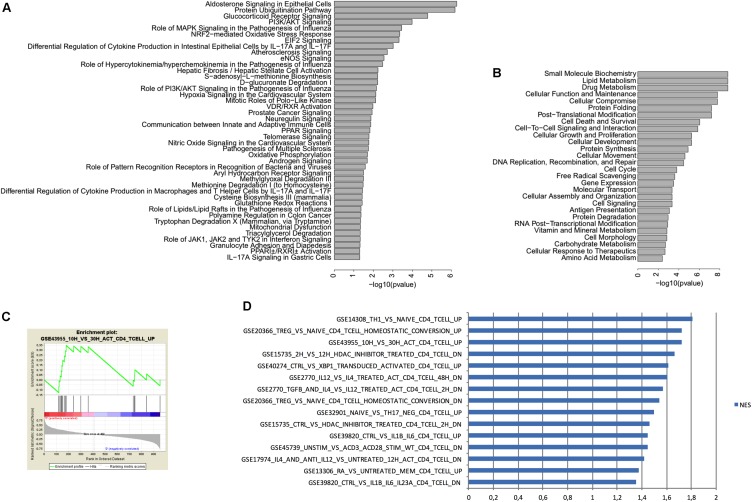
IPA and GSEA analysis of genes expressed in CD4^+^ T cells from the choroid plexus from EE mice vs. SE mice. **(A)** IPA Canonical pathways associated with differentially expressed genes (i.e., increased or decreased) when comparing CD4^+^ T cells between enriched and standard environments. We used IPA to compare the variation in gene expression observed in CD4^+^ T cells between enriched and standard environments (increase or decrease) to the gene variations observed in canonical pathways. The more significant pathways are indicated and bars represent the strength of the statistical significance. **(B)** IPA Molecular functions associated with differentially expressed genes (i.e., increased or decreased) when comparing CD4^+^ T cells between enriched and standard environments. We used IPA to compare the variation in gene expression observed in CD4^+^ T cells between enriched and standard environments (increase or decrease) to the gene variations associated with molecular functions. The more significant molecular functions are indicated and bars represent the strength of the statistical significance. **(C)** Example of an enrichment plot obtained using GSEA by comparison of the CD4^+^ T cells from the plexus (enriched vs. standard environment) to the C7 immunology set of data. **(D)** Top pathways obtained in GSEA for genes enriched in CD4^+^ T cells from the choroid plexus (EE mice *vs.* SE mice) with the higher NES. We considered only the pathways containing in their names “CD4” but not “CD8” or “transfected”.

To determine which pathways are involved in the differences between peripheral as well as brain CD4^+^ T cells from mice raised in EE vs. SE, we performed an Ingenuity Pathway Analysis (IPA) with the following parameters: *p* < 0.05, Log2 FC 0.5 and intensity 10. This analysis revealed that the genes with differential expression between SE and EE conditions were particularly involved in the glucocorticoid receptor-signaling pathway (**Figures 5A,B**).

We thus used GSEA to compare the overall changes induced between EE and SE housing conditions in the CD4^+^ T cells from choroid plexus, to previous data already published by others from the immunology set (C7, which uses only experiments performed on immune cells). We selected the pathways that corresponded to the variations observed in EE *vs.* SE with a *p* < 0.05 and sorted them according to the normalized enrichment score. We selected only experiments that used the same cell type (i.e., CD4^+^ T cells) by filtering according to the name of the experiments (**Figures 5C,D**). Overall, the gene expression profile obtained suggests that CD4^+^ T cells from choroid plexus in EE animals have an activated phenotype as compared to those from SE mice that presented a naïve profile.

As a conclusion, EE modifies gene expression in CD4^+^ T cells from the choroid plexus and their profiles might differ slightly as compared to SE.

## Discussion

In this study, we show that CD4^+^ T cells play a major role in EE-induced changes in hippocampus synaptic plasticity. The transcriptome of CD4^+^ T cells sorted from choroid plexus differed slightly in EE compared to SE. CD4^+^ T cells located in the choroid plexus, by their strategic position close to the hippocampus, could thus influence hippocampal plasticity.

We found that CD4^+^ T cell depletion reduced neurogenesis in the DG of the hippocampus both in SE and EE conditions. This is consistent with the reduction in neurogenesis in SE, which has already been described by others (Schwartz and Kipnis, 2011; Kipnis et al., 2012). It is interesting to note that CD4^+^ T cell depletion affects global neurogenesis but the increase in neurogenesis induced by EE is largely preserved in CD4^+^ T cell-depleted mice. This is different from our previous findings concerning CD8^+^ T cells, where their depletion fully blunted the increase in neurogenesis induced by EE (Zarif et al., 2018). Therefore, each sub-population of T cells plays a specific role in the control of neurogenesis.

On the other hand, CD4^+^ T cell depletion fully reversed the synaptic changes induced in CA1 pyramidal neurons by EE, as already described in (Zarif et al., 2018). These changes were in accordance with the variations we measured by RT-qPCR in the dissected CA of the hippocampus. We found that CD4^+^ T cell depletion reversed or delayed the increase in expression of genes coding for proteins that play a major role in the establishment of the glutamatergic synapse. This was true both at the presynaptic (synapsin 2, synaptophysin and synaptogamin) and at the post-synaptic levels (Dlg4, coding for the PSD95 scaffolding protein located in neural postsynaptic densities that associates with NMDA receptor NR2 subunits; homer1, encoding for Homer 1, a protein concentrated in postsynaptic structures; and Slc17a7 coding for the glutamate transporter Vglut1), and also concerned Bdnf.

These data were also in accordance with the electrophysiological recordings we performed in CA1 pyramidal neurons, as CD4^+^ T cell depletion impaired the increase in spontaneous (s) and miniature (m, recorded in the presence of TTX) synaptic activities in EE, compared to SE. Indeed, we show that an HFS leads to an LTP of smaller amplitude in mice raised for 4 weeks in EE compared to mice raised in SE, probably due to a ceiling effect as most synapses at 4 weeks have already a high level of potentiation in EE conditions (Hosseiny et al., 2015) but this difference was no longer observed in CD4^+^ T cell-depleted mice.

Overall, CD4^+^ T cells appear to play a major role in the synaptic plasticity processes occurring in hippocampus during EE. The present results are in accordance with data showing that SCID mice (devoid of both T and B cells) and nude mice (deficient in mature T cells) present learning deficiencies in the water maze, a hippocampal-dependent spatial learning and memory task (Kipnis et al., 2004; Ron-Harel et al., 2008). Impairment in learning and memory of SCID mice was also observed in three other tests involving hippocampal function – the radial arm water maze, the water-free Barnes maze (Brynskikh et al., 2008) and novel spatial arrangement recognition of familiar objects (Ron-Harel et al., 2008).

Under physiological conditions, lymphocytes do not enter the cerebral parenchyma but are strategically located at the interfaces between blood and CNS, potentially allowing them to act as environmental sensors and to modulate by physical or chemical mediation the microglial, astrocytic, and neuronal activities. This hypothesis implies at least two conditions: firstly, T cells must be, in one way or another, modified by their environment and secondly, their absence must influence communication systems between the periphery and the brain.

Regarding the first point, although no obvious changes were observed in the phenotype of peripheral CD4^+^ T cells in EE compared to SE, the RNAseq experiments showed that CD4^+^ T cells located in the choroid plexus undergo subtle changes in the expression of a set of genes in the brain under EE conditions relative to SE conditions. For instance, in CD4^+^ T cells from the choroid plexus, one of the top pathways identified by the IPA analysis was the glucocorticoid receptor pathway, which is consistent with the elevations in corticosterone observed in EE (Cao et al., 2010). In addition, GSEA analysis revealed that CD4^+^ T cells present a profile similar to that of memory T cells in the brain.

A large proportion of T cells in the brain are located in the choroid plexus. The choroid plexus are made of an epithelial barrier that separates the blood from the CSF contained in the ventricles. The lateral and third ventricles line the hippocampus, which makes T cells located in their choroid plexus possible strategic contributors to influence hippocampal plasticity. We speculate that CD4^+^ T cells found in the choroid plexus influence (either by direct contact or indirectly by secreting factors) the transport of molecules through the choroid plexus epithelium than can then reach the CSF and the brain parenchyma. Some factors produced by CD4^+^ T cells in EE such as cytokines or chemokines, or adiponectin, which has been shown to be produced by these cells in under certain conditions (Crawford et al., 2010; Danturti et al., 2017), could favor brain plasticity (Nicolas et al., 2015; Zhang et al., 2016). In this line, we found that genes encoding for CXCR6, IFNγ and CCL5 were upregulated in CD4^+^ T cells from choroid plexus of EE mice relative to SE mice. In addition, we found (not shown) that CD4^+^ T cell depletion affects in EE the expression of genes involved in the epithelial choroid plexus barrier and transport function, such as claudin and ttr, which could be the consequence of an imbalance in these factors normally produced by CD4^+^ T cells. Future investigations will decipher the putative role of these factors in EE-induced hippocampus plasticity.

Overall, our results suggest that CD4^+^ T cell participate to the regulation of EE-induced hippocampal plasticity.

## Author Contributions

HZ conceived and participated to all the experiments, did the figures, and participated in writing the paper. SH initiated the work on neurogenesis and performed some of the neurogenesis and electrophysiology experiments. M-JA did the RNAseq experiment. AP and KL analyzed the RNAseq data. JuC performed the cell sorting experiments. AL helped with the CD4^+^ T cell depletion protocol. CH helped in writing the paper. NG participated in conceiving the immunology experiments. AG participated in the electrophysiology experiments. JoC, AG, and AP-P conceived the experiments and wrote the paper.

## Conflict of Interest Statement

The authors declare that the research was conducted in the absence of any commercial or financial relationships that could be construed as a potential conflict of interest.

## Supplementary Material

FIGURE S1 Anti-CD4 antibody treatment is sufficient to eliminate all CD4^+^ T cells. Plots representing blood CD4/CD8 profile gated on CD45^+^ CD3^+^ population 3 days after an *ip* injection either of an anti-rat isotype antibody (control condition, left) or anti-CD4 (right).

FIGURE S2 Spine morphology in pyramidal neurons of CA1. **(A)** Schema of the different spine types. **(B)** Repartition of different dendritic spine types.

FIGURE S3 Histogram showing the mean number of cells per slice (2 slices labeled per hippocampus, *n* = 4 mice per group): BrdU^+^ cells and BrdU^+^ Doublecortin (DCX)^+^ cells **(A)** and BrdU^+^ Iba1^+^ cells **(B)** labeled in the DG.
