# Supplementary figures and images for: CD4+ T Cells Have a Permissive Effect on Enriched Environment-Induced Hippocampus Synaptic Plasticity
Source: Front Synaptic Neurosci. 2018 Jun 13;10:14. doi: 10.3389/fnsyn.2018.00014 (PMC6008389; doi:10.3389/fnsyn.2018.00014)

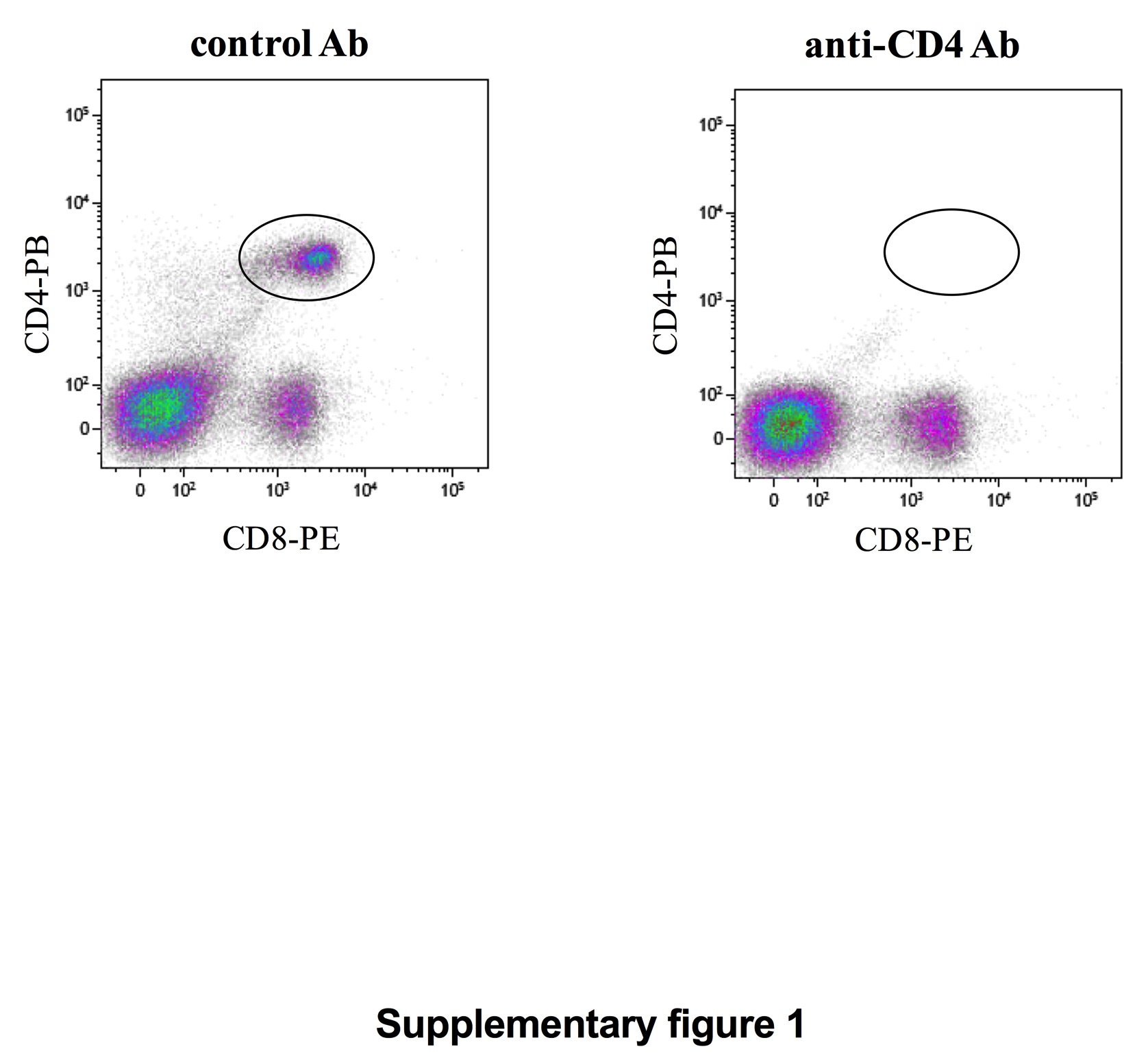

Supplement: FIGURE S1 — Anti-CD4 antibody treatment is sufficient to eliminate all CD4+ T cells. Plots representing blood CD4/CD8 profile gated on CD45+ CD3+ population 3 days after an ip injection either of an anti-rat isotype antibody (control condition, left) or anti-CD4 (right). [file Image_1.TIFF]

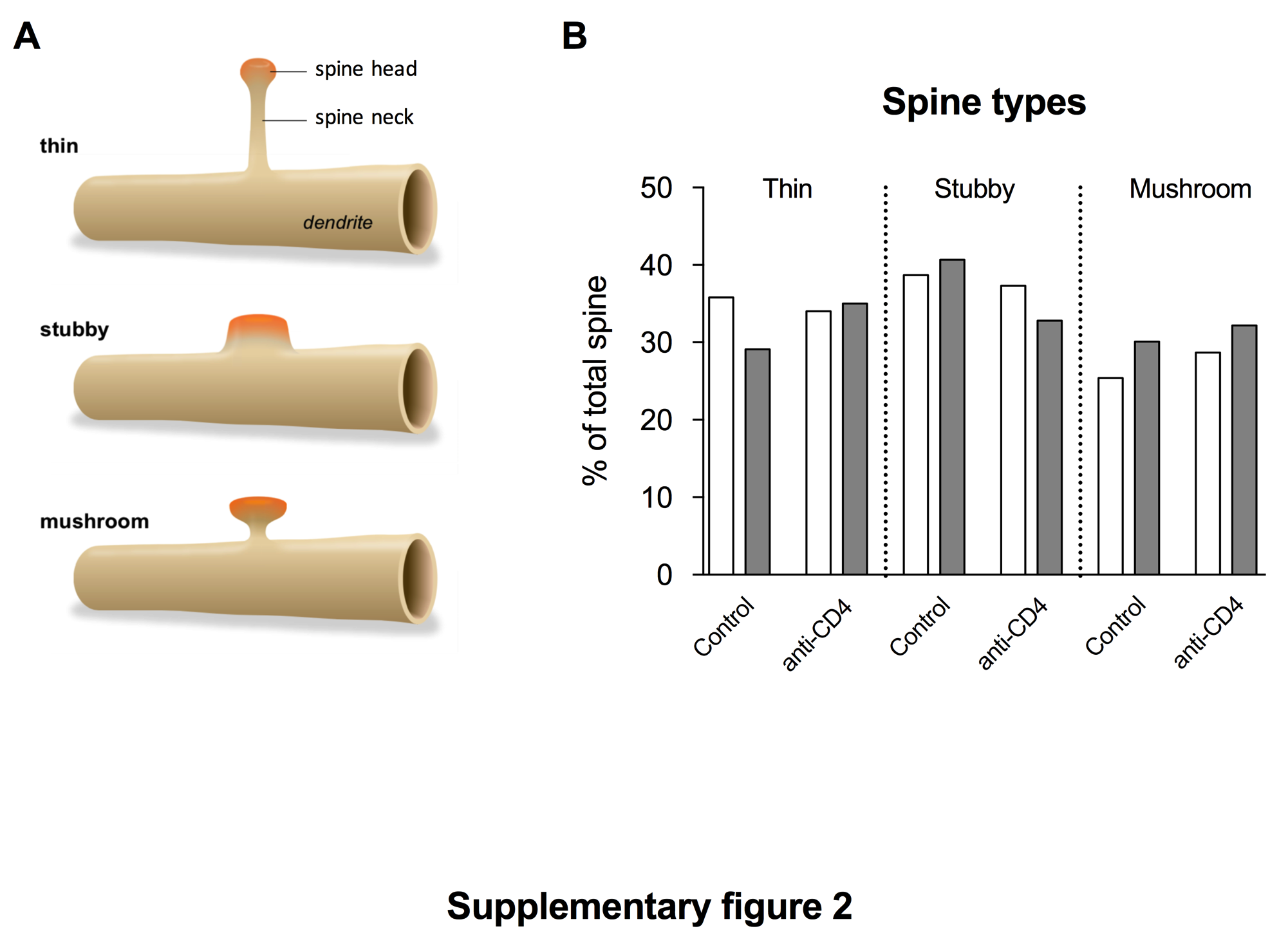

Supplement: FIGURE S2 — Spine morphology in pyramidal neurons of CA1. (A) Schema of the different spine types. (B) Repartition of different dendritic spine types. [file Image_2.TIFF]

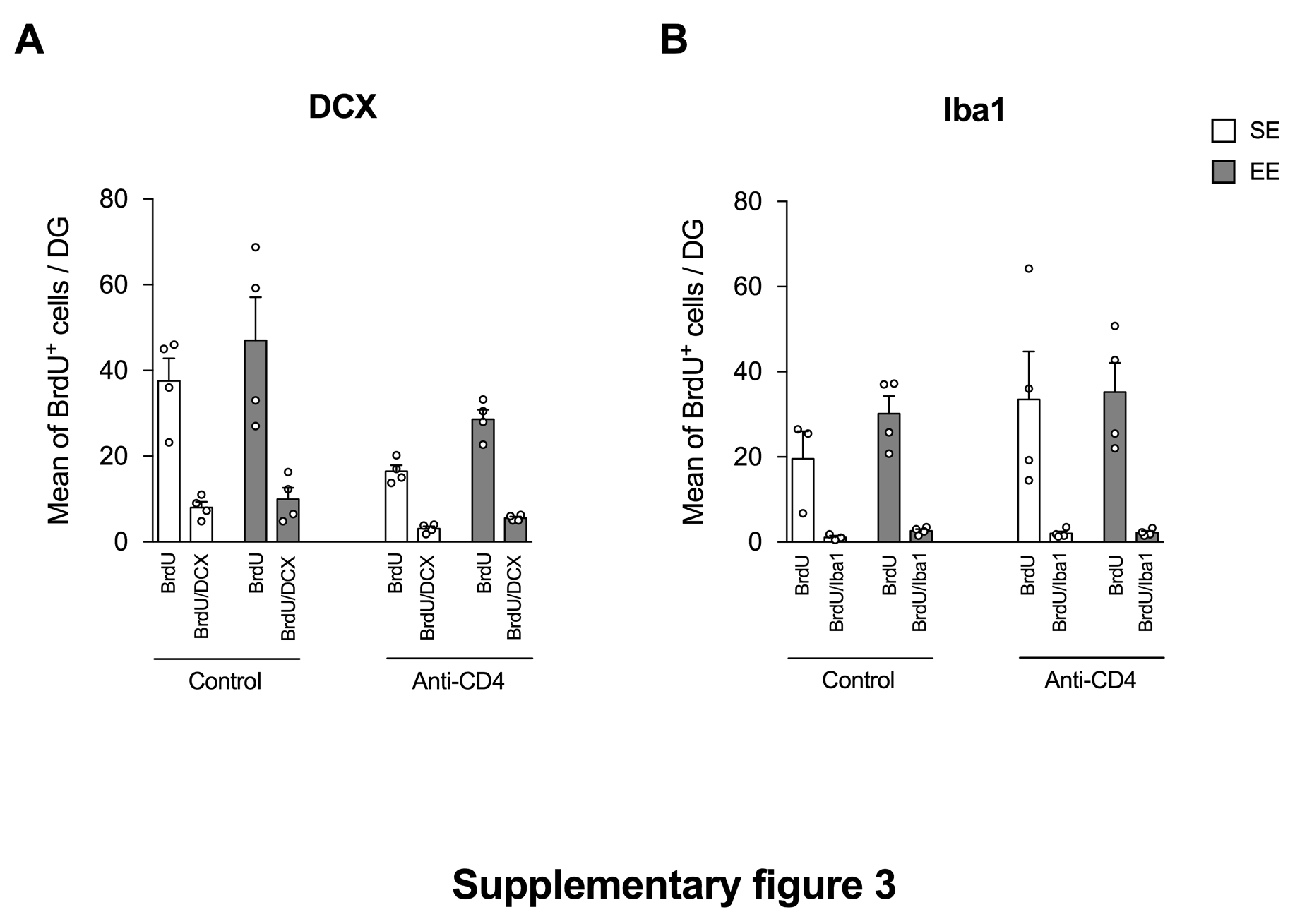

Supplement: FIGURE S3 — Histogram showing the mean number of cells per slice (2 slices labeled per hippocampus, n = 4 mice per group): BrdU+ cells and BrdU+ Doublecortin (DCX)+ cells (A) and BrdU+ Iba1+ cells (B) labeled in the DG. [file Image_3.TIFF]
